# Effects of different microplastics on the physicochemical properties and microbial diversity of rice rhizosphere soil

**DOI:** 10.3389/fmicb.2024.1513890

**Published:** 2025-01-21

**Authors:** Sheng Lai, Cunzhong Fan, Ping Yang, Yuanyuan Fang, Lanting Zhang, Minfei Jian, Guofei Dai, Jutao Liu, Huilin Yang, Liqin Shen

**Affiliations:** ^1^Jiangxi Academy of Water Science and Engineering, Nanchang, China; ^2^College of Life Science, Jiangxi Normal University, Nanchang, China; ^3^Jiangxi Provincial Technology Innovation Center for Ecological Water Engineering in Poyang Lake Basin, Nanchang, China

**Keywords:** microplastics, biodegradability, aging, physicochemical properties, rhizosphere, microorganisms

## Abstract

Biodegradable plastics, as alternatives to conventional waste plastics, are increasingly applied across various fields. However, the ecological risks associated with the widespread use of biodegradable plastics remain unclear. Additionally, biodegradable plastics tend to age in the environment, leading to changes in their physicochemical properties. The ecological risks brought by the aging of microplastics have also been scarcely studied. In this study, we selected conventional microplastics (PE-MPs), biodegradable microplastics (PLA-MPs), and aged biodegradable microplastics (aging-PLA-MPs) to explore their effects on the rhizosphere soil environment of rice. The results showed that microplastics reduced the soil N and P content, with PE slightly increasing the DOC content, while PLA and aging-PLA significantly increased DOC by 21.13 and 24.04%, respectively. Microplastics also decreased soil enzyme activity, with aging-PLA having a somewhat stimulatory effect on enzyme activity compared to PLA. Furthermore, microplastics reduced the soil bacterial diversity index and altered the community structure of dominant bacterial species, with DOC content and FDA hydrolase being the main factors influencing the soil bacterial community. Bacteria were most sensitive to PLA, and the stability of the bacterial microbial network structure decreased, although aging reduced the negative impact of PLA on the bacterial community. This study contributes to our understanding of the ecological risks posed by biodegradable plastics and their aging processes on the environment.

## Graphical abstract

PE, polyethylene microplastics; PLA, polylactic acid microplastics; APLA, aged polylactic acid microplastics; C, dissolved organic carbon; N, total nitrogen; P, available phosphorus; SC, soil sucrase; CAT, soil catalase; UE, soil urease; FDA, fluorescein diacetate.
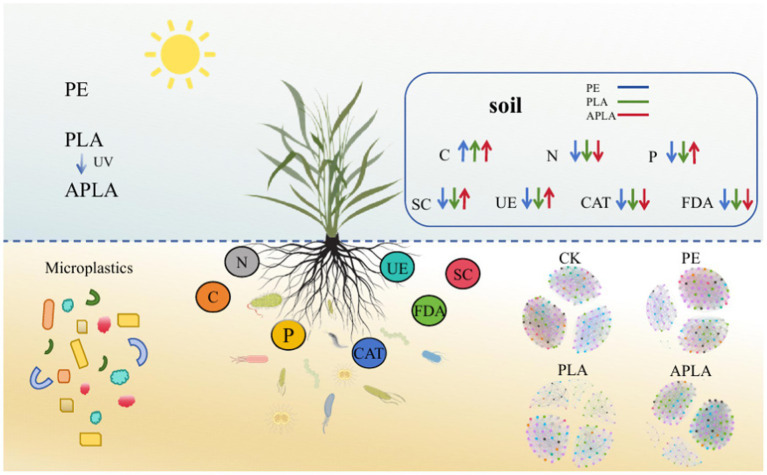

## Highlights

- Exposure to three kinds of microplastics decreased soil nutrient content and soil enzyme activity in rice roots.
- Three types of microplastics reduced bacterial microbial diversity and changed soil microbial community structure.
- Biodegradable microplastics (polylactic acid microplastics) are more toxic to soil systems than conventional microplastics (polyethylene microplastics).
- Aging reduces the negative effects of polylactic acid microplastics on soil.

## 1 Introduction

Discarded plastics can break down and wear away under natural conditions, eventually fragmenting into tiny pieces or particles that become widely dispersed in the atmosphere, water bodies, and soil, significantly impacting natural ecosystems (Kumar et al., 2024; Zendehboudi et al., 2024). When the particle size of these plastic fragments is less than 5 mm, they are defined as microplastics (MPs) (Thompson et al., 2004). There is evidence that terrestrial environments are not only sources and sinks for microplastics but also the primary sources of plastic waste in the oceans. The extent of microplastic pollution in terrestrial environments may be 4 to 23 times greater than in marine environments (Luo et al., 2021). Microplastics in the soil can alter soil properties and structure, potentially causing adverse effects on soil fauna, plants, and microorganisms, thereby posing a potential threat to terrestrial ecosystems.

Once microplastics enter the soil, their effects span multiple aspects, including the soil itself, as well as plant and microbial communities (Okeke et al., 2022). They primarily impact the formation of soil aggregates, leading to changes in soil porosity, bulk density, and permeability, which are crucial to the soil environment and its ecological functions (Wan et al., 2019; Ya et al., 2021). Zhou et al. (2024) found that the binding of microplastics with soil aggregates led to a reduction in soil bulk density by more than 15%, and a decline in crop growth performance. Similarly, research by de Souza Machado et al. (2018) showed that microplastics affect soil porosity and the interactions between soil particles, which in turn influence soil aggregates, nutrient content, and enzyme activity. This impact likely arises from the large specific surface area of microplastics, as they adsorb key substrates of soil enzymes and compete with soil microorganisms for ecological niches, thereby inhibiting microbial activity and the function of soil enzymes (Yu et al., 2020). Microplastics not only directly affect soil enzyme activity but also indirectly influence the circulation of oxygen and water within the soil by altering its physicochemical properties. These changes can further lead to shifts in the abundance of anaerobic and aerobic microorganisms, thus altering microbial community structures (Rubol et al., 2013; Wang F. et al., 2022). Additionally, microplastics may release carbon sources, which can impact the composition and function of carbon- and nitrogen-cycling microbial communities in the soil (Rillig, 2018).

Biodegradable plastics are those that can degrade under the action of certain microorganisms, such as algae, bacteria, or fungi, naturally present in the environment (Vadillo et al., 2023). Currently, biodegradable plastics are gradually replacing conventional plastics and are widely used in various fields, including food packaging, agriculture, and healthcare (Shen et al., 2019; Taib et al., 2023). However, some studies suggest that biodegradable microplastics may interfere with soil nutrient cycling and disrupt microbial activity, potentially posing even greater risks than conventional microplastics (Iqbal et al., 2020). Pedram Jarf et al. (2024) found that biodegradable microplastics release phthalates during decomposition in the soil, and these additives can disrupt the existing structure of soil bacterial communities. Additionally, research by Wang et al. (2020) demonstrated that the chemical toxicity generated during the degradation of PLA-MPs adversely affects the diversity and community structure of arbuscular mycorrhizal fungi. Increasing evidence indicates that biodegradable microplastics may trigger more complex ecological effects.

Aged microplastics reflect the combined effects of environmental and intrinsic factors. Aging refers to the process by which the physicochemical and biological properties of plastics change due to physical abrasion, UV radiation, and biodegradation in natural environments. Microplastics undergo continuous aging under various environmental factors, leading to an increase in their specific surface area, enhanced hydrophilicity, and an increase in oxygen-containing functional groups, which in turn affect their original physical properties (Huang et al., 2021). After aging, the surface roughness of microplastics increases, significantly altering their adsorption properties, making them more prone to adsorb pollutants (Fries et al., 2013; Mammo et al., 2020). For instance, Bhagat et al. (2021) found that aging is a key factor in the adsorption of heavy metals, with aged microplastics showing enhanced adsorption of soil organic matter and heavy metals, potentially posing more severe threats to soil ecology through coupled pollution (Kutralam-Muniasamy et al., 2021; Torres et al., 2021).

Research by Wang et al. (2021) demonstrated that microplastics altered the bioavailability of cadmium in the soil, leading to increased cadmium accumulation in lettuce and exacerbating damage to plants. Moreover, the additives in microplastics, such as plasticizers and flame retardants added during industrial processing, also have significant effects. These chemicals gradually release into the environment and may interact with microorganisms, plants, and animals in the soil. The aging process accelerates the release of these toxic substances, leading to unknown environmental toxicity and ecological risks (Barrick et al., 2021; Hahladakis et al., 2018; Hermabessiere et al., 2017). The aging process of microplastics under natural ecological conditions varies, and research on microplastic aging remains scarce. Further investigation is warranted to understand the mechanisms of microplastic aging and their interactions with other substances in the environment.

It is well known that rice is a crucial staple crop in China and worldwide. Galahitigama et al. (2024) reviewed how MPs trigger oxidative stress in rice plants, causing changes at morphological, physiological, biochemical, metabolic, and molecular levels. In addition, MPs influence the rice rhizosphere by modifying soil properties, microbial diversity, and metabolic processes. Mbachu et al. (2021) indicates that the accumulation of micro- and nanoplastics (MNPs) in terrestrial ecosystems induces significant changes in various soil parameters. These include alterations in physicochemical properties such as soil nutrients, aggregation, porosity, bulk density, water saturation capacity, pH, and cation exchange capacity (CEC) following exposure to microplastics. Therefore, it is important to consider the effects of microplastics on plant growth media. Rhizosphere soil refers to the portion of soil directly influenced by plant roots. Being in close proximity to the roots, its physicochemical properties have a direct impact on plant growth (Gao et al., 2020). Moreover, the rhizosphere soil also develops a microbial ecosystem with specific structures and functions (Hester et al., 2018).

Previous studies have often overlooked the impact of microplastics on the rhizosphere soil environment of plants after entering the soil. Considering the current state of microplastic research and advancements in biodegradable microplastic studies, we selected conventional microplastics, biodegradable microplastics, and aged biodegradable microplastics to investigate their effects on rice rhizosphere soil properties and microbial communities. By measuring changes in soil nutrient content and enzyme activity under different microplastic treatments, combined with high-throughput sequencing to analyze the effects of microplastics on soil microorganisms, we explored the influence of microplastics on the abiotic and biotic components of the rice rhizosphere. This study aims to clarify the impact of different types of microplastics on the rhizosphere soil environment of rice.

We proposed the following hypotheses: (1) the presence of microplastics affects the rhizosphere soil environment of rice; (2) the biodegradability of microplastics may result in different impacts on the rhizosphere soil environment; (3) UV aging alters the morphology and properties of microplastics, and this accelerated degradation process could also lead to variations in the rhizosphere soil environment. The study’s findings will provide a better understanding of the ecological risks posed by microplastics and contribute to exploring the feasibility of replacing conventional materials with biodegradable alternatives in agricultural systems. Our study aims to better understand the ecological risks posed by microplastics. The results will contribute to assessing the feasibility of replacing conventional materials with biodegradable alternatives in agricultural systems.

## 2 Materials and methods

### 2.1 Soil sampling and microplastics

**Figure 1:**
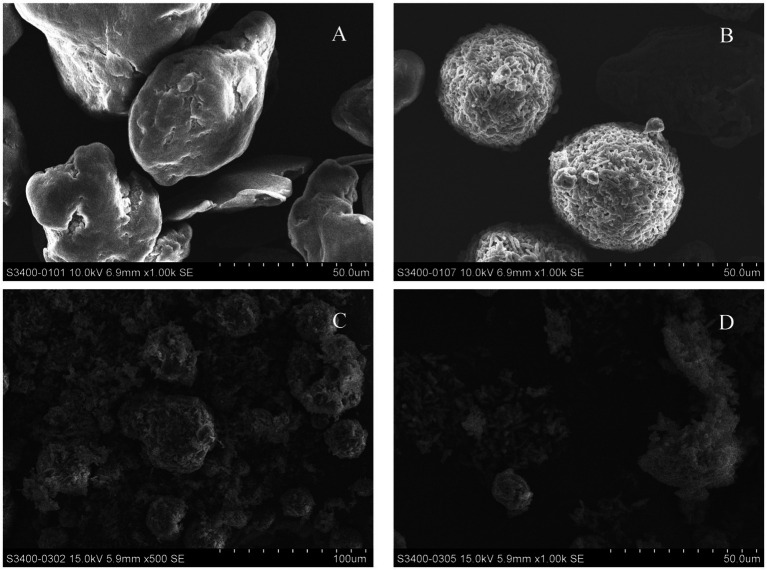
Morphology of different microplastics under scanning electron microscopy. **(A)** Polyethylene microplastics at 1000× magnification. **(B)** Polylactic acid microplastics at 1000× magnification. **(C)** Aged polylactic acid microplastics at 500× magnification. **(D)** Aged polylactic acid microplastics 1,000× magnification.

The soil used in this experiment was collected from the labor practice base of Jiangxi Normal University. The soil type is sandy loam, the area has a subtropical monsoon climate with an average annual temperature of 16°C–24°C. After natural air-drying, impurities such as stones and tree roots were removed, and the soil was sieved through a 2 mm stainless steel mesh for later use. The common rice variety Nanbran 5718 was selected as the research object and purchased from Suqian Xiwen Seed Co., Ltd., Jiangsu Province. Three types of microplastics were selected as test materials for this experiment: polyethylene microplastics (PE-MPs), polylactic acid microplastics (PLA-MPs), and aged polylactic acid microplastics (aged-PLA-MPs). PE-MPs were chosen because they are among the most widely polluting plastics, while PLA-MPs represent biodegradable plastics. The aged-PLA-MPs were created by subjecting PLA-MPs to UV aging (a 253.7 nm UV light source was used for 12 h per day for 1 month). The microplastics were purchased from Zhangmutou Ruixiang Polymer Materials Co., Ltd., Dongguan. The morphology and size of the MPs were characterized using scanning electron microscopy (SEM). Figure 1 shows PE at 1000× magnification (Figure 1A), PLA at 1000× magnification (Figure 1B), and APLA at 500× and 1,000× magnifications (Figures 1C,D). The morphological differences between PE-MPs and PLA-MPs are evident, with the former having a smoother surface and the latter appearing rougher. Figures 1C,D show that UV exposure causes significant surface cracking on APLA-MPs, which is distinct from the surface characteristics of PLA-MPs.

### 2.2 Experimental design

To simulate future microplastic pollution in farmland soil, the dry weight concentration of microplastics in the soil was set at 1%, with a particle size range of 35–48 μm. We chose concentrations higher than the actual environmental pollution levels mainly considering that microplastics are not uniformly distributed in agricultural fields. For example, in areas where fragments of agricultural film are concentrated, the microplastic content may be relatively high. For another, microplastics within this size range are more prone to environmental dispersion and present a higher environmental risk. The experiment included a blank control group with no added microplastics and three treatment groups: PE-MPs, PLA-MPs, and aged-PLA-MPs, designated as CK, PE, PLA, and APLA, respectively. Each treatment group was replicated three times. Rice seeds with the same degree of germination were selected and cultivated in a constant temperature plant growth chamber (14 h/25°C during the day and 10 h/14°C at night) with a light intensity of 3,000 lx and a relative humidity of 70% (Zhou et al., 2021). After 4 weeks of rice cultivation, the experiment was concluded, and rhizosphere soil was collected. The rice plants were carefully shaken to protect their root systems, with subsequent removal of loose soil surrounding them. The soil adhering closely to the roots was then collected as the rhizosphere soil sample (Razavi et al., 2017; Zhu et al., 2022). One portion of the soil was air-dried under natural conditions for the determination of soil nutrient content and enzyme activity, while another portion was stored at −80°C for the extraction of soil bacterial DNA.

### 2.3 Determination of soil physicochemical properties

The soil pH value was measured using a pH meter (Coulter Bech-man Co., United States). Total nitrogen content in the soil was determined using the Kjeldahl method (Kjeltec 2100, CHN) (Cui et al., 2020; Sáez-Plaza et al., 2013). 0.5 g of air-dried soil were combined with NaOH in a sealed container to facilitate the hydrolysis of nitrogen-containing compounds and the release of NH₃ gas. A boric acid-indicator solution was placed above the reaction mixture to absorb the NH₃ during a 24-h incubation at 50°C. Following the incubation period, the absorbed NH₃ was quantitatively measured through titration. The nitrogen content in the soil was determined based on the volume of HCl utilized in the titration process (Lu, 1999). Available phosphorus (AP) were extracted with 0.5 M NaHCO_3_ (Olsen, 1954). The extracts of AP were filtered through a Millipore 0.45-μm filter. The contents of AP were then determined by the molybdenum blue method using an ultraviolet spectrophotometer (Hitachi UV2300). Appropriate amount of 0.5 M K₂SO₄ was added to 0.5 g soil sample, and after shaking on a shaker for 60 min, the extract was filtered. TOC analyzer (hed-sz-002, CHN) was used to determine the content of dissolved organic carbon in the extracts (Ghani et al., 2003).

### 2.4 Assessment of soil enzyme activities

Soil sucrase activity was determined using the 3,5-dinitrosalicylic acid colorimetric method. Urease activity was measured using the sodium hypochlorite-sodium phenolate colorimetric method. Peroxidase activity was assessed using the o-phenylenediamine colorimetric method. FDA hydrolase activity was measured using ultraviolet spectrophotometry. All measurements were conducted using kits purchased from Suzhou Geruisi Biotechnology Co., Ltd.,^1^ and the procedures were followed according to the instructions provided in the kit manuals (Zhou et al., 2020).

Soil sucrase breaks down sucrose into reducing sugars, which react with 3,5-dinitrosalicylic acid to form colored compounds measurable at 540 nm. Sucrase activity is defined as the enzyme amount needed to produce 1 mg of glucose per gram of soil per day. Urease hydrolyzes urea to release NH₃-N, which forms indophenol blue in an alkaline medium with phenol and hypochlorite. This dye absorbs light at 578 nm, and urease activity is the enzyme amount required to produce 1 μg of NH₃-N per gram of soil per day. Catalase decomposes H₂O₂ into water and oxygen. Residual H₂O₂ reacts with a chromogenic probe to form a compound absorbing at 510 nm. Catalase activity is the enzyme amount needed to degrade 1 μmol of H₂O₂ per gram of soil per hour. Fluorescein diacetate (FDA) is hydrolyzed by various enzymes to produce fluorescein, which absorbs strongly at 490 nm. FDA activity is defined as the enzyme amount producing 1 μg of fluorescein per gram of soil per hour.

### 2.5 DNA extraction, PCR amplification and sequencings library construction

DNA extraction was performed using a Soil Microbial Genomic DNA Extraction Kit (Fujian Fuzhou) with 0.5 g of fresh soil. The quality of the extracted DNA was assessed by 1% agarose gel electrophoresis. The extracted DNA was used as a template for PCR amplification with barcode-specific primers. The V4 region of the 16S rRNA gene was amplified with 515F (5′-GTGYCAGCMGCCGCGGTAA-3′) and 806R (5′-GGACTACNVGGGTWTCTAAT-3′) primers (Zhang et al., 2022). The PCR was carried out with a total volume of 20 μL: DNA template 10–100 ng, KOD FX Neo Buffer: 10 μL, 515F (10 mM): 0.5 μL, 806R (10 mM): 0.5 μL, KOD FX Neo 0.4 μL, dNTP (2 mM each) 4 μL and ddH_2_O up to 20 μL. Thermal cycling conditions were 94°C for 2 min followed by 28 cycles of 98°C for 10 s, 55°C for 30 s, and 68°C for 30 s, and a final extension at 68°C for 7 min. PCR products from the same sample were analyzed by 1% agarose gel electrophoresis, and purified using the Gel Extraction Kit (Omega). The purified DNA was then quantified using a Nanodrop 2000 spectrophotometer and normalized. Finally, the constructed libraries were used with Illumina NovaSeq 6000 (Illumina, Santiago CA, United States) for sequencing at Biomarker Technologies Corporation, Beijing, China. The purified PCR products were prepared for sequencing libraries using the NEXTFLEX Rapid DNA-Seq Kit, which involved adapter ligation, removal of self-ligated adapter fragments through bead-based selection, enrichment of library templates via PCR amplification, and final recovery of the PCR products using magnetic beads to obtain the completed library.

### 2.6 High-throughput sequencings data analysis

Raw FASTQ files were demultiplexed using a custom Perl script, followed by quality filtering with fastp (v0.19.6) (Chen et al., 2018) and sequence merging with FLASH (v1.2.7) (Magoč and Salzberg, 2011) under the following conditions: (i) reads of 300 bp were truncated at sites with an average quality score below 20 within a 50 bp sliding window, and those shorter than 50 bp or containing ambiguous bases were discarded; (ii) only overlapping sequences longer than 10 bp were merged based on their overlap, allowing a maximum mismatch ratio of 0.2 in the overlap region. Non-mergeable reads were excluded; (iii) samples were identified using barcode and primer sequences, allowing exact barcode matches and up to 2 mismatches in primer matching, with sequence direction adjusted accordingly. The filtered sequences were clustered into operational taxonomic units (OTUs) at 97% similarity using UPARSE (v7.1) (Edgar, 2013; Stackebrandt and Goebel, 1994), with the most abundant sequence in each OTU designated as the representative. Taxonomic classification of OTU representatives was performed using the RDP Classifier (v2.2) (Wang et al., 2007) against the 16S rRNA gene database (e.g., Silva v138) with a confidence threshold of 0.7.

### 2.7 Statistical analysis

The soil environmental factors and microbial indicators were analyzed using one-way ANOVA and least significant difference (LSD) tests with IBM SPSS Statistics 21 software to assess the significance of differences. Results are presented as mean ± standard deviation. Alpha diversity metrics, such as ACE and Shannon indices, were calculated using the mothur (Schloss et al., 2009) software.^2^ Differences in alpha diversity between groups were analyzed using the Wilcoxon rank-sum test. Principal coordinate analysis (PCoA) based on the Bray–Curtis distance algorithm was employed to assess the similarity of microbial community structures between samples, and PERMANOVA was used to determine whether the differences in microbial community structure among sample groups were statistically significant. Microbial community co-occurrence network analysis was conducted using the “psych” package in R 4.2.0, based on Spearman’s correlation coefficient, and visualized using Gephi 0.9.2 software. Correlation analysis between soil environmental factors and microorganisms was performed using the Biomarker BioCloud platform.^3^

## 3 Result

### 3.1 Effects of different microplastics on soil physicochemical properties

We observed significant changes in some soil nutrient indicators (Table 1). Specifically, soil dissolved organic carbon (DOC) levels generally increased under microplastic exposure, particularly with biodegradable microplastics (PLA and APLA), where soil DOC content significantly increased by 21.13 and 24.04% (*p* < 0.05). Additionally, microplastic exposure resulted in a reduction in total nitrogen (TN) and alkaline hydrolysable nitrogen (AN) content in the soil; notably, soil TN decreased significantly by 16.98% under PE-MPs exposure. The soil nutrient content was higher under APLA-MPs exposure compared to the PLA group. This suggests that aged biodegradable microplastics may have a more positive effect on soil nutrient retention and enhancement.

**Table 1:** Effects of different microplastics on soil sample nutrients.

Treatments	pH	DOC (mg/kg)	TN (g/kg)	AN (mg/kg)	AP (mg/kg)
CK	7.99 ± 0.15a	55.41 ± 4.27b	0.53 ± 0.03a	21.34 ± 3.22a	283.79 ± 16.74ab
PE	7.92 ± 0.20a	57.06 ± 3.41b	0.44 ± 0.03b	17.05 ± 2.83a	257.91 ± 13.66b
PLA	7.83 ± 0.13a	67.12 ± 4.80a	0.48 ± 0.05ab	18.13 ± 2.13a	274.13 ± 21.89ab
APLA	7.87 ± 0.10a	68.73 ± 5.57a	0.49 ± 0.04ab	18.28 ± 3.07a	304.88 ± 19.68a

DOC, dissolved organic carbon; TN, total nitrogen; AN, alkaline hydrolysable nitrogen; AP, available phosphorus; S_UE, soil urease. Values with different lowercase letters are significantly different (*p* < 0.05).

### 3.2 Effects of different microplastics on soil enzyme activity

The changes in enzyme activities under different microplastic treatments are shown in Table 2. Exposure to PE-MPs and PLA-MPs significantly reduced soil sucrase (S-SC) activity by 33.17 and 10.66%, respectively, while exposure to APLA-MPs slightly increased soil sucrase activity. Soil urease (S-UE) activity decreased significantly by 15.32 and 40.38% under PE-MPs and PLA-MPs exposure, respectively; although APLA-MPs exposure increased urease activity, the effect was not significant. Soil catalase (S-CAT) activity decreased significantly by 19.11 and 50.91% under PE-MPs and APLA-MPs exposure, respectively. Microplastics significantly reduced soil FDA hydrolase activity, with PE-MPs, PLA-MPs, and APLA-MPs exposure resulting in decreases of 29.56, 68.13, and 34.43%, respectively. Notably, soil FDA hydrolase activity in the APLA group was 105.80% higher than that in the PLA group. Among the soil enzymes exposed to aged microplastics, all except peroxidase showed higher activity compared to the original biodegradable microplastics.

**Table 2:** Effects of different microplastics treatments on soil enzyme activities.

Treatments	S-SC	S-UE	S-CAT	S-FDA
CK	8.35 ± 0.39a	54.04 ± 2.64a	177.99 ± 8.06a	50.34 ± 2.05a
PE	5.58 ± 0.28c	45.76 ± 2.78b	143.97 ± 16.16b	35.46 ± 2.19b
PLA	7.46 ± 0.33b	32.22 ± 2.29c	165.79 ± 17.14a	16.04 ± 2.30c
APLA	8.74 ± 0.39a	57.62 ± 3.78a	87.38 ± 6.24c	33.01 ± 2.18b

S-SC, soil sucrase; S-UE, soil urease; S-CAT, soil catalase; FDA, fluorescein diacetate. Values with different lowercase letters are significantly different (*p* < 0.05).

### 3.3 Effect of different microplastics on soil microbial diversity

After exposure to microplastics, the α-diversity indices of soil bacteria are shown in Table 3. Alpha diversity describes the biodiversity within a specific region or ecosystem, typically characterized by calculating diversity indices based on species richness or evenness. The ACE index is commonly used to estimate the total species count within a community, while the Shannon and Simpson indices reflect both species richness and evenness. These indices provide a relatively objective measure of community species diversity. Microplastic exposure reduced soil bacterial α-diversity indices. Specifically, the α-diversity indices in the PLA group were significantly lower than those in other treatments, indicating that PLA-MPs had the greatest impact on soil bacterial α-diversity. The ACE, Shannon, and Simpson indices in the APLA group were higher compared to the PLA group, suggesting that aging of microplastics enhanced microbial diversity.

**Table 3:** Alpha diversity index of soil bacteria under different microplastics treatment.

Treatments	ACE index	Shannon index	Simpson index
CK	1062.67 ± 270.93a	8.87 ± 0.26a	0.99 ± 0.00a
PE	670.00 ± 159.22b	8.16 ± 0.50a	0.99 ± 0.00a
PLA	160.05 ± 81.04c	6.04 ± 0.70c	0.97 ± 0.01b
APLA	323.00 ± 79.88c	7.00 ± 0.14b	0.98 ± 0.00a

The ACE index is used to estimate the total number of species within a community, the Shannon index and Simpson index reflect both species richness and evenness. Values with different lowercase letters are significantly different (*p* < 0.05).

**Figure 2:**
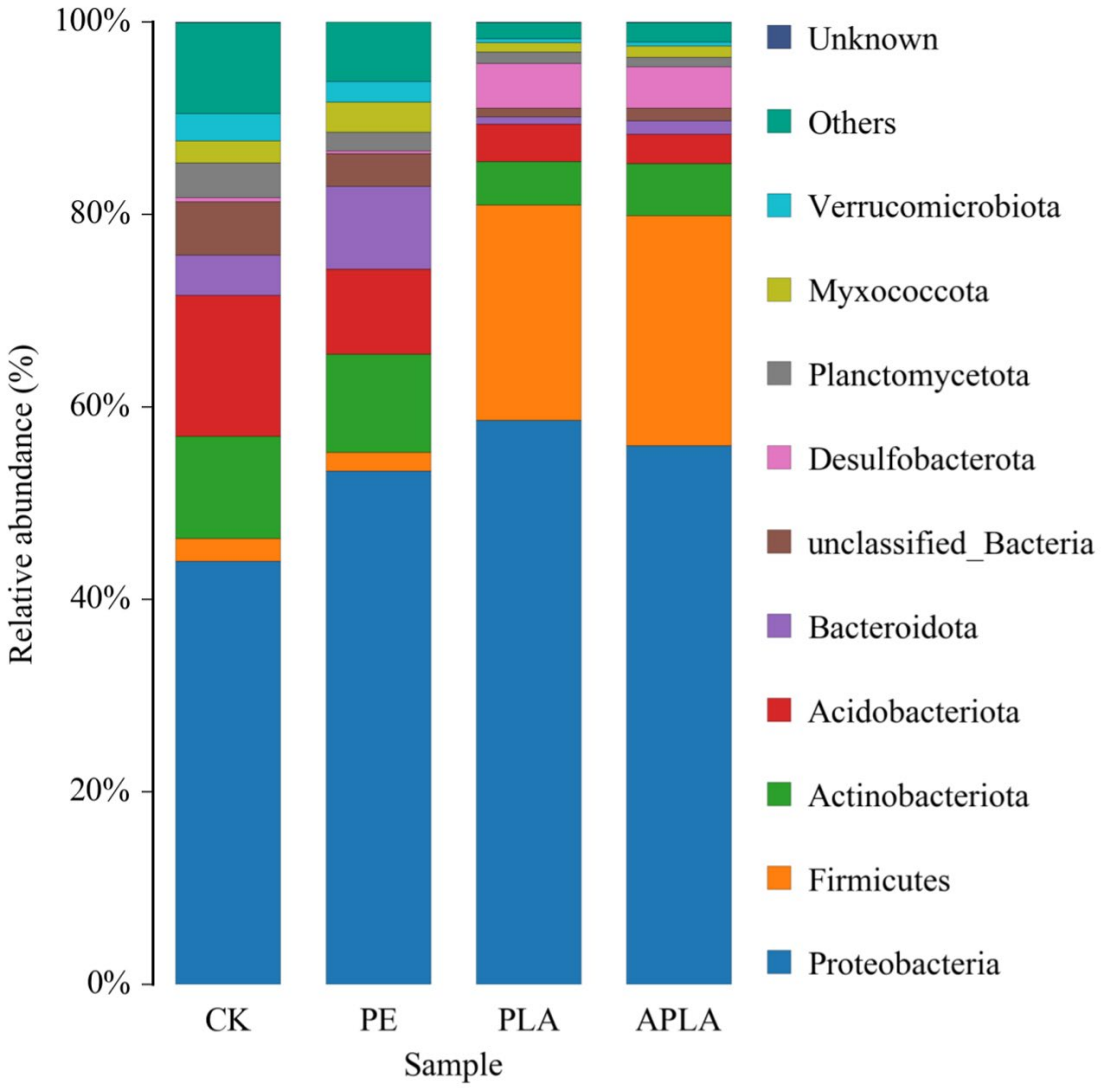
Soil bacterial community composition at phyla level. CK, control check group without microplastics; PE, soil with polyethylene microplastics; PLA, soil with polylactic acid microplastics; APLA, soil with aged polylactic acid microplastics. “Other” refers to the species that are not included in the top 10 based on relative abundance and are grouped together. “Unknown” refers to species that have not yet been isolated or identified.

The relative abundances of the 10 bacterial phyla at the phylum level in the soil bacterial community are shown in Figure 2. At the phylum level, Proteobacteria was the dominant bacterial phylum across all treatments, the relative abundance of Proteobacteria significantly increased in all treatments, with an increase of 9.38 to 14.66%. Among other bacterial phyla, compared to the CK group, the relative abundance of Acidobacteriota decreased by 5.82% in the PE group, while the relative abundance of Bacteroidota increased by 4.42%. In the PLA and APLA groups, the relative abundance of Firmicutes increased by 20.01 and 21.46%, respectively. Meanwhile, the relative abundance of Actinobacteriota decreased by 6.10 and 5.16%, Acidobacteriota decreased by 10.74 and 11.60%, and Desulfobacterota increased by 4.27 and 3.90%, respectively.

**Figure 3:**
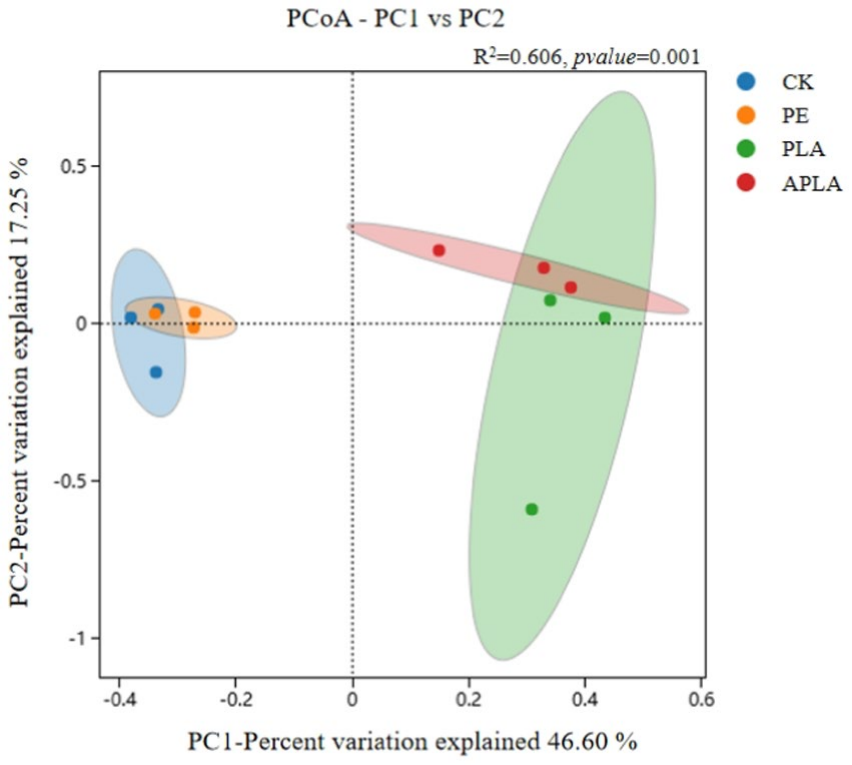
Cluster analysis of PCoA community structure of soil bacteria. Confidence intervals are plotted at a 95% confidence level, representing the distribution range of each sample and the differences between different treatments. *R*^2^ = 0.606, *p*-value = 0.001.

**Figure 4:**
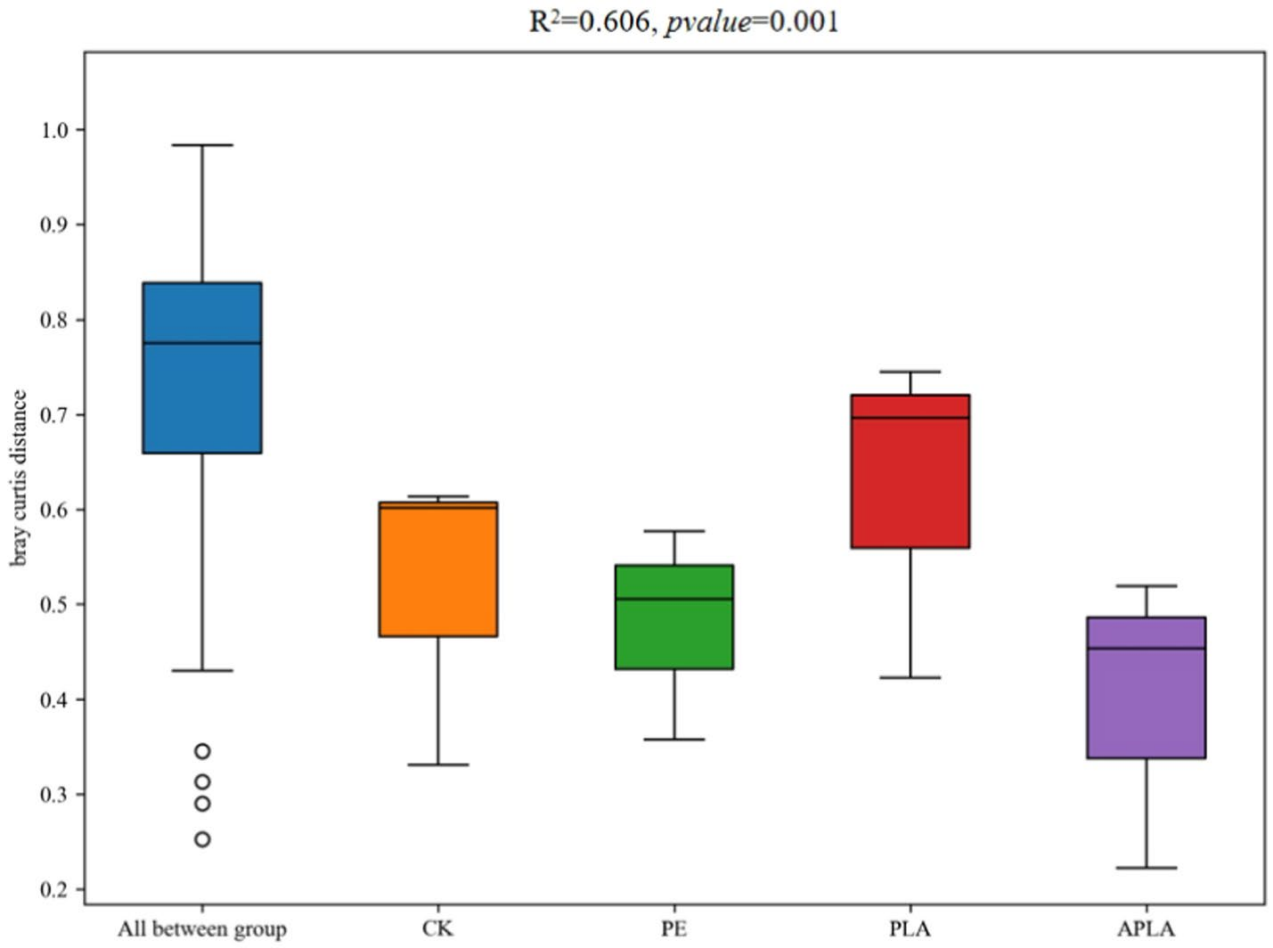
PERMANOVA analysis of community structure of soil bacteria. Bray–Curtis algorithm is used to reflect the differences in microbial community structure among sample groups. Different colors represent different microplastic treatments. *R*^2^ = 0.606, *p*-value = 0.001.

β-diversity primarily refers to the diversity between sample groups and reveals differences in bacterial community structure in rice soil under different microplastic treatments. As shown in Figure 3, principal coordinates analysis (PCoA) was performed to classify the samples and distinguish differences in species diversity among them. For bacterial communities, PC1 and PC2 explained 46.60 and 17.25% of the variance, respectively, contributing a total of 63.85% (*R*^2^ = 0.606, *p* = 0.001). Additionally, permutational multivariate analysis of variance (PERMANOVA) was used to further elucidate microbial community diversity (Figure 4). This method evaluates the influence of grouping on differences, with higher *R*^2^ values indicating a greater explanatory power for group differences and more significant group variation. Soil bacterial communities under different microplastic treatments clustered strongly, with similar species composition within groups and significant differences between groups.

**Figure 5:**
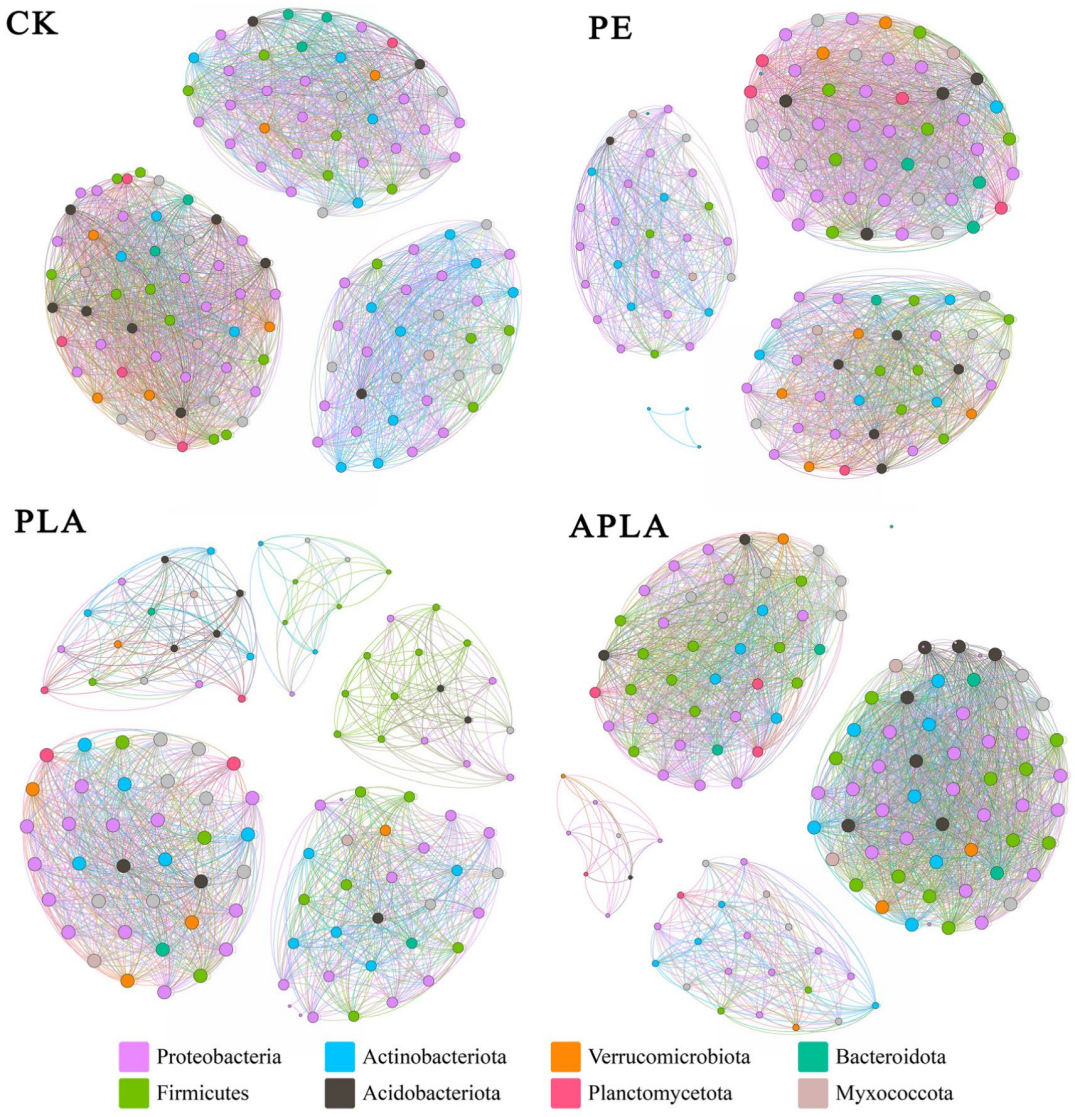
Co-occurring network model of soil bacteria and microorganisms. Different colored nodes represent different microbiota phylum, and the lines between nodes represent the connections among these microbial populations. CK, control check group without microplastics; PE, soil with polyethylene microplastics; PLA, soil with polylactic acid microplastics; APLA, soil with aged polylactic acid microplastics.

The microbial co-occurrence network structure can reveal the interaction relationships between soil microbial communities under different microplastic treatments. Bacterial microbes with a relative abundance higher than 0.1% at the genus level were selected to construct the microbial co-occurrence network diagrams. Under different microplastic treatments, the microbial communities formed distinct network structures (Figure 5). In the CK, PE, PLA, and APLA groups, the soil bacterial co-occurrence networks had 126, 122, 110, and 129 nodes, and 2,610, 2,432, 1,449, and 2,624 edges, with average degrees of 41.429, 39.869, 26.345, and 40.682, respectively. The positive correlation coefficients were 60.34, 60.4, 62.39, and 55.18%, while the negative correlation coefficients were 39.66, 39.6, 37.61, and 44.82%, respectively. In the PLA group, the bacterial network had fewer nodes, edges, and a lower average degree compared to other treatments, indicating that PLA-MPs reduced the network scale and connectivity of soil bacteria. The APLA group had more nodes and edges than the other treatment groups, but also had a higher negative correlation coefficient. This suggests that APLA-MPs enhanced the interconnections among soil microorganisms, but weakened the cooperative interactions among them and increased competitive interactions between populations. The aging treatment increased the complexity of the bacterial network and mitigated the negative effects of microplastics on soil microbial diversity.

### 3.4 Correlation analysis

**Figure 6:**
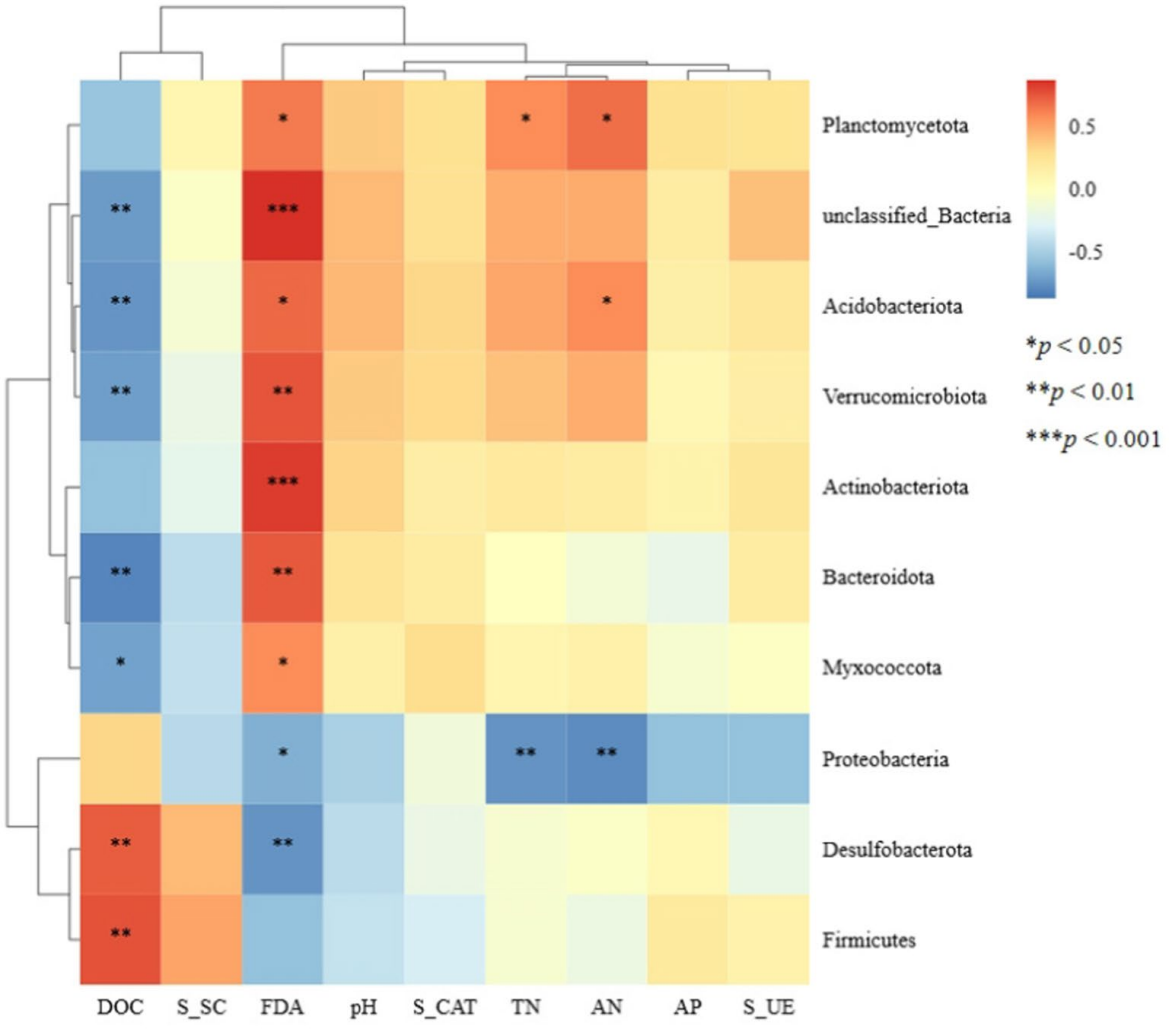
Heat maps of the correlation between bacterial microorganisms and environmental factors based on phylum level. DOC, dissolved organic carbon; S_SC, soil sucrase; FDA, fluorescein diacetate; S_CAT, soil catalase; TN, total nitrogen; AN, alkaline hydrolysable nitrogen; AP, available phosphorus; S_UE, soil urease. ^*^p < 0.05, ^**^p < 0.01, and ^***^p < 0.001.

Spearman correlation analysis was conducted between the top 10 bacterial phyla relative abundances and soil physicochemical properties as well as soil enzyme activities (Figure 6). At the phylum level, the relative abundances of Planctomycetota, unclassified_Bacteria, Acidobacteriota, Verrucomicrobiota, Bacteroidota, and Myxococcota were significantly positively correlated with soil FDA hydrolase activity and significantly negatively correlated with soil dissolved organic carbon (DOC) content. The relative abundances of Desulfobacterota and Firmicutes were significantly positively correlated with soil DOC content. In contrast, the relative abundance of Proteobacteria was significantly negatively correlated with soil nitrogen content and FDA hydrolase activity.

## 4 Discussion

Soil dissolved organic carbon (DOC) is crucial for soil biogeochemical cycles due to its high mobility and bioavailability. Liu et al. (2017) found that low doses of microplastics reduced DOC content, whereas high doses of microplastics activated carbon pools, increasing soil DOC levels. Sun et al. (2022) assessed the impact of biodegradable microplastics versus conventional microplastics on soil DOC, revealing that biodegradable microplastics significantly increased soil DOC compared to conventional microplastics. This increase is attributed to the organic polymer nature of microplastics, which are more likely to decompose in the soil and release carbon sources, thereby raising DOC levels (Lee et al., 2020). Additionally, the enrichment of microorganisms utilizing biodegradable microplastics contributes to the rise in DOC content, as these microbes are involved in the degradation and transformation of microplastics in the soil. Similar findings were also reported by Wang F. et al. (2022).

Previous studies have shown that microplastics can alter the complexity of C-N coupling in soil-plant systems. One contributing factor is the input of microplastics themselves as a carbon source. Additionally, Xiao et al. (2019) reported that the presence of microplastics downregulates plant N-related genes, reducing the transport of nitrogen to the aboveground parts of plants. Nitrogen deficiency forces plants to allocate more carbon belowground to meet their nutritional demands. Similarly, Hu et al. (2024) found that photosynthesized carbon may be released into the soil through root rhizodeposition, potentially increasing soil carbon levels. This aligns with our findings of elevated dissolved organic carbon (DOC) levels in soils treated with microplastics.

Microplastics also directly reduce soil nitrogen content, affecting nitrogen cycling (Seeley et al., 2020). Qian et al. (2018) attributed this primarily to the inhibitory effects of microplastics on the expression of soil nitrogen cycling genes. Microplastics can interfere with microbial activity, altering soil nitrogen content by impacting microbial respiration and nitrification processes, thereby affecting N_2_O and CO_2_ emissions (Iqbal et al., 2020; Wan et al., 2023). Our study observed increased carbon content and decreased nitrogen content under microplastic treatments, resulting in higher C/N ratios. Specifically, treatments with PE-MPs, PLA-MPs, and APLA-MPs increased the C/N ratio by 24.64, 34.68, and 35.40%, respectively, with significant effects observed for biodegradable microplastics. Alterations in the soil C/N system could pose unpredictable risks to agricultural productivity and ecosystem stability by limiting the availability of essential nutrients for microbes and plants, potentially leading to excessive greenhouse gas emissions and other unforeseen consequences (Iqbal et al., 2024).

Wang et al. (2024) found that soil available phosphorus content decreased by 9.7 to 38.6% and 38.4 to 73.6% under exposure to conventional microplastics (PE, PVC) and biodegradable microplastics (PLA), respectively, across three different soil types. Research indicates that aging affects the adsorption properties of microplastics. Initially, PLA-MPs primarily adhere to soil through physical adsorption, but aged PLA-MPs involve both physical and chemical adsorption mechanisms (Yu et al., 2023). Aging enhances the adsorption capacity of microplastics because the surface structure of aged microplastic fragments becomes more porous, providing additional ion adsorption sites. Based on these findings, we anticipated that soil available phosphorus content would be lower under APLA-MPs exposure. However, the APLA group exhibited the highest soil available phosphorus content. This suggests that although aging increases the adsorption effect of microplastics, this enhanced adsorption effect is insufficient to decrease available phosphorus levels. We hypothesize that the increase in soil available phosphorus is primarily due to the release of organic acids, metal chelators, and other intermediate products during the degradation of APLA-MPs.

Soil enzymes serve as crucial indicators for assessing soil biological activity and health, with their activity reflecting the direction and intensity of biochemical processes occurring in the soil (Hagmann et al., 2015; Wei et al., 2017). Research indicates that urease activity is correlated with soil available nitrogen, and microplastics may alter soil nitrogen content by inhibiting urease activity (Dong et al., 2021). Consistent with previous analyses, the introduction of microplastics into the soil interferes with microbial activity, thereby affecting urease secretion and indirectly influencing soil nitrogen content. Our study found a significant reduction in soil sucrase activity in the PE treatment group, which aligns with findings by Yang et al. (2021), who observed that low concentrations of PE-MPs with three small particle sizes (<25 μm, 25–48 μm, 48–150 μm) decreased soil sucrase activity. They attributed this effect to the impact of microplastics on soil microbial activity, which in turn reduced sucrase activity, a conclusion also supported by Yu et al. (2021). Microplastic exposure affects soil pH, electrical conductivity (EC), bulk density, and porosity, which subsequently alters the habitat conditions for sucrase activity in the soil (Wang F. et al., 2022). Additionally, changes in soil nutrient content due to microplastic introduction may also impact enzyme survival and hydrolytic processes. Under APLA-MPs exposure, soil enzyme activity was enhanced, likely due to the morphological changes in aged microplastics that more effectively stimulate soil microbial activity, thereby promoting enzyme activity to some extent.

Catalase (S-CAT) primarily originates from soil microorganisms and root exudates of plants, with its main function being the detoxification of accumulated hydrogen peroxide in the soil, thereby minimizing damage to plant roots (Duan et al., 2018). The results of this study indicated that all types of microplastics reduced soil catalase activity. However, in the study conducted by Huang et al. (2019), microplastics were found to increase catalase activity. This discrepancy may be attributed to differences in microplastic particle size and soil type used in the experiments. Further investigation suggests that soil catalase activity can serve as an indicator of aerobic microbial activity, which is closely related to the abundance of aerobic microorganisms in the soil (Liu et al., 2017). Toward the end of the experiment, soil compaction was observed, likely reducing oxygen flux within soil pores and adversely affecting the growth and proliferation of relevant microorganisms. This is likely a primary reason for the observed decline in catalase activity. Among the three types of microplastics, aged APLA-MPs may have degraded into smaller particles, leading to a higher degree of soil compaction, which further decreased catalase activity.

FDA hydrolase is a crucial indicator of organic matter transformation and microbial activity levels within soil systems, with its activity being closely correlated with microbial dynamics. Exposure to microplastics generally leads to a reduction in soil FDA hydrolase activity (Fei et al., 2020), with the extent of the impact varying in the following order: PLA > APLA > PE. PLA-MPs have the most significant effect on reducing soil FDA hydrolase activity, which is consistent with the findings of this study regarding microbial diversity indices, highlighting the strong relationship between FDA hydrolase activity and microbial activity. Currently, research on the effects of biodegradable microplastics on soil FDA hydrolase is still relatively limited. It is hypothesized that the decline in FDA hydrolase activity could be attributed to the degradation of PLA-MPs during incubation, which leads to the release of additives contained within the microplastics, subsequently exerting toxic effects on soil microbes and thereby reducing enzyme activity. Additionally, SEM observations reveal that APLA-MPs fragment into finer particles, which may increase their availability to microorganisms. This enhanced microbial access could stimulate the growth of microbes that utilize these microplastics as a carbon source, potentially explaining why the FDA hydrolase activity in the APLA group is higher than in the PLA group.

Soil microbial diversity, richness, and community structure are critical indicators of soil health and quality (Ji et al., 2014; Shen et al., 2016). Generally, higher microbial community richness and diversity, along with a more complex community structure, suggest greater stability of the microbial community. Microplastic exposure significantly impacts microbial communities, reducing soil bacterial richness (Wang Q. et al., 2022). Furthermore, microplastics can alter soil microbial communities by providing surfaces that enrich microplastic-degrading microorganisms, such as Acidobacteriota, Bacteroidota, and Chloroflexi. These bacterial communities may utilize carbon sources from microplastic degradation to meet their metabolic needs, leading to marked differences from other soil environments (Huang et al., 2019). Polyethylene microplastics (PE-MPs), being resistant to degradation, do not directly interact with microorganisms but instead act as external substrates that selectively alter the structure of certain microbial communities (Zhang et al., 2019). In contrast, polylactic acid microplastics (PLA-MPs), as biodegradable plastics, may have more complex effects on microbial communities in the soil environment. Unlike conventional microplastics, PLA-MPs not only provide a new habitat for microbes but also introduce additional physical and chemical impacts on the soil through their degradation process (Kong et al., 2018).

In terms of microbial community structure, the relative abundance of Proteobacteria significantly increased, while that of Actinobacteriota notably decreased. This shift could be attributed to microplastics altering soil bulk density and reducing soil aeration. The decrease in oxygen diffusion favors the growth of Proteobacteria, which are typically obligate or facultative anaerobes. By the end of the experiment, the soil in the PLA and APLA groups exhibited higher compaction and lower oxygen flux compared to the PE group, leading to a higher abundance of Proteobacteria in these treatments. This confirms that the addition of microplastics alters soil physicochemical properties, thereby influencing the soil microbial community. Furthermore, the Firmicutes increased in abundance, while Acidobacteriota decreased in the PLA and APLA groups. Studies have shown that Firmicutes typically proliferate rapidly in high-carbon environments (Irshad et al., 2024). PLA-MPs, being organic polymers, contribute carbon sources to the soil, which is one reason Firmicutes became a dominant phylum in these groups. The Desulfobacterota consisting of anaerobic bacteria, also increased in relative abundance in the PLA and APLA groups. This is because Desulfobacterota primarily utilize sulfur compounds as electron acceptors, conducting sulfur metabolism under low-oxygen or microaerobic conditions. The reduced oxygen flux in PLA and APLA soils favored the growth of Desulfobacterota. Additionally, some research suggests that Desulfobacterota have the ability to degrade microplastics, utilizing them as substrates for growth and metabolism, which may also explain their increased abundance in the PLA and APLA groups.

Microbial co-occurrence networks are instrumental in understanding the interrelationships within soil microbial communities, with more complex bacterial networks indicative of a more stable microecological environment. Our study found that the presence of microplastics alters soil bacterial diversity and community structure, with bacterial communities exhibiting higher sensitivity to PLA-MPs. PLA-MPs significantly reduced the connectivity among bacterial communities, weakening microbial interactions, thereby affecting network complexity and stability. However, the bacterial network in soils treated with aged PLA-MPs (APLA-MPs) showed greater stability compared to native PLA-MPs. This enhanced stability may be due to the increased bioavailability of aged microplastics, which stimulated the growth of microbial communities reliant on carbon sources. The competitive growth of these microbial communities also led to lower positive correlation coefficients within the bacterial co-occurrence network in the APLA group. Correlation analysis revealed that most dominant bacterial phyla were significantly associated with soil dissolved organic carbon (DOC) content and FDA hydrolase activity, as DOC serves as a substrate for bacterial proliferation, and FDA hydrolase is closely related to microbial activity. Based on our findings, microplastics reduce bacterial diversity and alter community structure; however, as a carbon source, microplastics may also facilitate microbial growth and reproduction. The aging process exacerbates these effects, highlighting the need for future research to consider the ecological impacts of environmental aging on microplastics.

## 5 Conclusion

Soil is a complex and heterogeneous environment, and the introduction of microplastics not only affects soil nutrients and enzyme activities but also influences microbial communities. This study examines the negative impacts of three types of microplastics on soil environments. As we hypothesized, the presence of microplastics can affect the rhizosphere soil environment of rice. Conventional microplastics (PE-MPs) primarily affect soil nutrients and enzyme activities while having a relatively minor impact on soil microbial communities. In contrast, biodegradable microplastics (PLA-MPs) exhibit some positive effects on soil nutrients but significantly alter the structure of soil microbial communities, likely due to the rapid release of internal additives during their degradation in the soil. Microplastic aging is also a critical factor influencing the soil environment. The aging process reduces the negative effects of microplastics on the soil, but the botanical effects resulting from the fragmentation of microplastics during aging remain uncertain. Previous studies have shown that nanoplastics pose more severe ecological risks than their micron-sized counterparts. The aging process may accelerate the transformation of microplastics into nanoplastics, though the connection between micron- and nano-sized plastics remains unclear. Future research should focus on evaluating the overall impact of microplastics on the “soil-plant” system across different plant species. This approach will help us better understand the broader ecological consequences of microplastics.

## Data Availability

The datasets presented in this study can be found in online repositories. The names of the repository/repositories and accession number(s) can be found at: https://ngdc.cncb.ac.cn/gsa, CRA019018.

## Footnotes

^1^ http://www.geruisi-bio.com/

^2^ http://www.mothur.org/wiki/Calculators

^3^ https://www.biocloud.net
